# Compact Shortwave Infrared Imaging Spectrometer Based on a Catadioptric Prism

**DOI:** 10.3390/s22124611

**Published:** 2022-06-18

**Authors:** Lei Feng, Xiaoying He, Yacan Li, Lidong Wei, Yunfeng Nie, Juanjuan Jing, Jinsong Zhou

**Affiliations:** 1 Key Laboratory of Computational Optical Imaging Technology, Aerospace Information Research Institute, Chinese Academy of Sciences, Beijing 100094, China; fenglei@aircas.ac.cn (L.F.); hxy@aoe.ac.cn (X.H.); lyc@aoe.ac.cn (Y.L.); weilidong@aoe.ac.cn (L.W.); jingjuanjuan@aoe.ac.cn (J.J.); 2 Department of Applied Physics and Photonics, Vrije Universiteit Brussel, 1050 Brussels, Belgium; 3 University of Chinese Academy of Sciences, Beijing 100049, China

**Keywords:** curved prism, infrared spectrometer, compact volume

## Abstract

This article demonstrates a compact prism imaging spectrometer method. A catadioptric curved prism is located at the secondary mirror position of the spectrometer and used to balance the aberrations, enlarge the dispersion width, and decrease the volume. A mathematical model of the prism and spectrometer is derived, which provides an optimal initial structure for a non-coaxial spectrometer, simplifying the optical design process and reducing the system volume. Using this method, a compact shortwave infrared imaging spectrometer with a 16° field of view is designed with an F-number/3, and the measured spectrum ranges from 0.95 to 2.5 μm. The performance is analyzed and evaluated. Laboratory testing results prove the excellent optical performance, and under the same specifications, the spectrometer length decreases by 40%.

## 1. Introduction

Imaging spectrometers can simultaneously acquire the spectral and spatial information of detected targets and are extensively used in various fields such as geology, biology, battlefield surveillance, and environment monitoring [1,2,3,4]. As several spectral absorption and reflective features exist across the shortwave infrared (SWIR) spectrum, the SWIR spectral band is important for many application domains including soil, vegetation, and false military targets covered by paint camouflage [5,6]. Compared to longwave infrared (LWIR) spectrometers, the SWIR counterparts mainly detect the radiation characteristics reflected by scenes [7]; however, their performance has been limited by detector technology, and the research on and development of SWIR imaging spectrometers have been inferior to those of LWIR and VNIR devices [8]. With the improvement of HgCdTe technology and breakthroughs in compound semiconductor material research [9,10], significant progress has been realized in SWIR detector technology, providing technical support for imaging spectrometers.

With the rapid development of optics and unmanned aerial vehicle technology [11], imaging spectrometers are required to be compact and have broad width, high resolution, and high signal-to-noise ratio (SNR). Early representative SWIR systems include the satellite-based main load EnMAP, the Hyperion, COIS, and AVIRS [12,13,14], and commercial systems developed by Headwall (USA) and Specim (Finland) [15,16,17]. The most common is the pushbroom imaging spectrometer [12], whose optical systems are of two main types: grating and prism-grating. Although grating-based imaging spectrometers have linear dispersion and are compact, the optical throughput is low because of the grating diffraction efficiency. Therefore, the main shortcoming of these systems is that compactness and high performance cannot be realized simultaneously [18]. Comparatively, prism-based spectrometers have advantages such as the absence of spectral overlap, high SNR, and low cost [19]. Prisms, including plane triangles and curved prisms, are dispersive elements, However, triangle plane prisms, such as Amici prisms, which are applied on parallel optical paths, only provide dispersion but not focusing. A curved prism is used as a prominent dispersion element for focusing and dispersion. Therefore, the realization of miniature prism-based spectrometers is of considerable value. Considering the application requirements and limitations of the available detectors, we research curved prism-based SWIR imaging spectrometers for airborne applications.

This report proposes a compact SWIR spectrometer method using a catadioptric curved prism and derives the corresponding mathematical model, as well as a simplified calculation method for the design process. A catadioptric curved prism is located at the secondary mirror position of the spectrometer, providing reflection once and dispersion twice. Based on the theory of minimum aberration and concentric matching, a mathematical model of the prism is derived that provides the relationship between the prism parameter and aberration, and a mathematical model of a prism-based spectrometer is established. This model can be used to provide a starting point for optimization and overcoming the difficulty of constructing the initial structure. This structure has several merits. First, by refracting the light twice, the dispersive width is increased. Moreover, the introduction of a catadioptric curved prism can balance the high-order aberrations correlated with increasing the incident angle caused by compacting the volume. Furthermore, the assembly space distance between the primary mirror and prism on the arm increases, which decreases the assembly difficulty. These advantages can be used to improve the performance, achieve compact configurations, and decrease the cost. Using the proposed method, a compact spectrometer prototype with a high SNR and large field-of-view (FOV) was designed and developed with a 0.95–2.5 μm spectral range, F-number of 3, and FOV of 16°. As the SWIR spectrum is invisible, the adjustment factors must be considered. We allocated moderate tolerance and investigated an assembly method to reduce the difficulty of adjusting the system. The remainder of this paper is organized as follows. In Section 2, the aberration theory of a curved prism is deduced, and the mathematical model is established. In Section 3, the opto-mechanical design and manufacture of the prototype are described. The performance tests and evaluation of the prototype are presented in Section 4. Finally, Section 5 concludes the paper.

## 2. Mathematical Model

The following mathematical model of the spectrometer can solve the problems related to the calculation of an appropriate initial structure for a curved prism-based spectrometer. Initially, the model of a curved prism is established based on the aberration theory. According to the theory of minimum aberration, the optimal object distance and incident angle of the curved prism are solved. Further, the curved prism-based spectrometer model is constructed. The incident and emitted vectors of the given ray are calculated using the vector solving method, and by combining the method of coordinate transform in a non-coaxial system and the ray-tracing of a tilted spherical surface, the intersection coordinates of a ray on the elements are obtained. Finally, the telescope is designed considering the achromatic balance and characteristics of SWIR optical materials. To improve the optical throughput of the system, the relative cold stop apertures are suitably matched.

### 2.1. Aberration Theory of Catadioptric Prism

In this study, a curved prism that can provide a certain focal power was used as the dispersive element. The spherical centers of the front and rear surfaces were not on the same optical axis, which disperses light.

**Figure 1:**
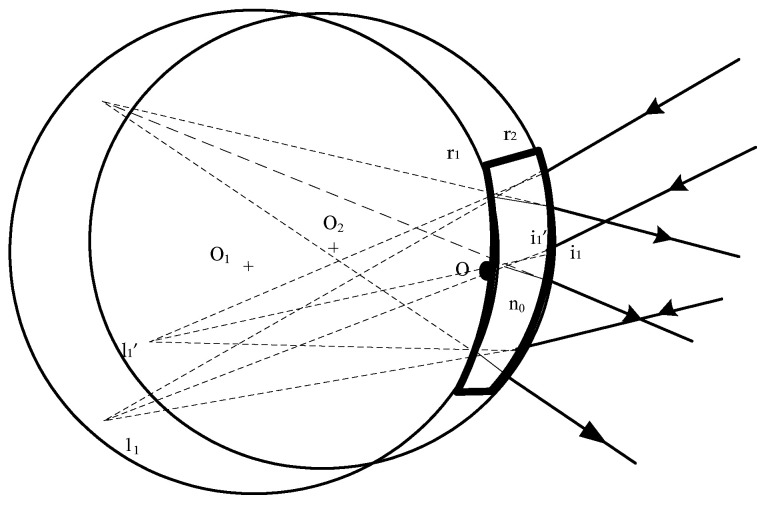
Model of a catadioptric prism.

In Figure 1, O is the vertex; 1_1_ and l_1_′ are the object and image distances relative to the vertex of the front spherical surface at a given point, respectively; the incident and emitted angles of the given incident ray are i_1_ and i_1_′, respectively; O_1_ and O_2_ are the centers of the front and back surfaces, respectively; r_1_ and r_2_ are the radii of the front and back surfaces, respectively; the dispersion index of the material is n_0_; and Δ_1_ and Δ_2_ are the off-axis values of the front and back surfaces, respectively. When a ray is incident on the front surface of the prism, the relationship between the object and image points of the emitted ray can be obtained based on the relation between the object and image:

(1) $$\frac{1}{r_{1}}-\frac{1}{l_{1}}=n_{0}(\frac{1}{r_{1}}-\frac{1}{l_{1}^{\prime }})$$

The image distance l1′of the emitted ray is given by

(2) $$l_{1}^{\prime }=\frac{n_{0}l_{1}r_{1}}{n_{0}l_{1}-l_{1}+r_{1}}.$$

Based on the three-order aberration theory, supposing that the off-axis value Δ_1_ is infinitely small, the primary Seidel coefficients can be deduced as follows [20]:

(3) $$\left\{ \begin{array}{l} S_{Ip1}=-\frac{1}{8}\frac{i_{1}^{\prime }}{i_{1}}*\Delta_{1}*n^{2}{\left(\frac{1}{r_{1}}-\frac{1}{l_{1}}\right)}^{2}(\frac{1}{n_{0}^{\prime }l_{1}^{\prime }}-\frac{1}{n_{0}l_{1}})\\ S_{IIp1}=\frac{i_{1}^{\prime }}{i_{1}}*\Delta_{1}*\frac{n_{0}^{2}}{2}(\frac{1}{r_{1}}-\frac{1}{l_{1}})(\frac{1}{n_{0}^{\prime }l_{1}^{\prime }}-\frac{1}{n_{0}l_{1}})\\ S_{IIIp1}=-i_{1}{}^{2}*\Delta_{1}*\frac{n_{0}^{2}}{2}(\frac{1}{n_{0}^{\prime }l_{1}^{\prime }}-\frac{1}{n_{0}l_{1}}) \end{array}\right.,$$

where S_IP1_, S_IIP2_, and S_IIIP1_ are the respective primary spherical, coma, and astigmatism coefficients of the given surface. To obtain the optimal object distance, a method of solving the approximation radii r_1_ = r_2_ = r is employed. According to the theory of minimum aberration, the specific solution can be obtained as follows:

(4) $$r=\frac{l_{1}}{n_{0}+1},\;r=l_{1}.$$

When the object is located at the homogeneous conjugate point or center of the spherical surface, the primary aberration is minimized. As shown in Figure 1, when a catadioptric prism is used in the converging optical path, the virtual object and image satisfy the Rowland principle, and one part of the aberrations can be compensated by itself, whereas another part can be used to balance the high-order aberrations of other elements.

### 2.2. Spectrometer Model

**Figure 2:**
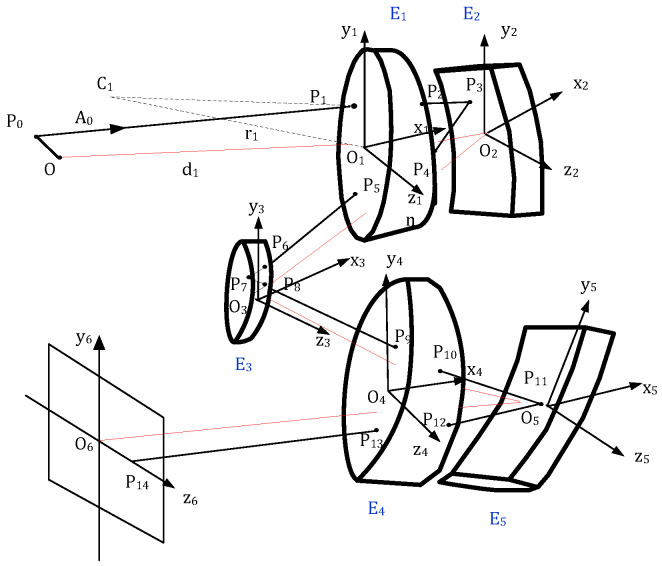
Dispersion diagram of a curved prism-based spectrometer.

The curved prism-based spectrometer is a non-coaxial symmetrical system shown in Figure 2. It is constructed by three curved prisms and two mirrors, where one prism is a catadioptric prism, which provides refraction, reflection, and dispersion, and the other two prisms are located on the two arms and mainly provide dispersion. The right-hand coordinate is the common coordinate, the *x*-axis coincides with the assembly optical axis of prism 1, and the vertex of the front spherical surface is considered as the origin. The position and direction vectors are used to describe the geometric position of any off-axis point. θ_1_ and θ_2_ are the surface tilt angles of prism 1, P_0_ is any given point located on the slit, and C_1_ is the center of the front surface. $\vec{P_{0}}$ is the incident position vector of a given point, and $\vec{A_{0}}$ is the unit direction vector. Pi (i = 0, 1, … 14) is the object point and the corresponding interception point on each surface; x_i_y_i_z_i_ (i = 1, 2 … 6) represents the coordinate system of different elements. Oi is the vertex of each element, r_1_ is the radius of the front surface of E_1_, and d_1_ is the distance between the slit and E_1_.

(5) $$\vec{P_{0}}(x,y,z)=x\vec{i}+y\vec{j}+z\vec{k},$$

(6) $$\vec{A_{0}}(\alpha ,\beta ,\gamma )=\alpha \vec{i}+\beta \vec{j}+\gamma \vec{k},$$

$P_{1}(x_{1},y_{1},z_{1})$ is located on the front spherical surface of prism 1 and is described in vector as follows:

(7) $$\vec{P_{1}^{2}}+2r_{1}\cos\theta_{1}\vec{i}\cdot \vec{P_{1}}+2r_{1}\sin\theta_{1}\vec{j}\cdot \vec{P_{1}}=0.$$

In triangle $\Delta O_{1}P_{1}C_{1}$,

(8) $$\vec{P_{1}}+r_{1}\vec{N_{1}}=-r_{1}\cos\theta_{1}\cdot \vec{i}-r_{1}\sin\theta_{1}\cdot \vec{j}$$

and

(9) $$\vec{N_{1}}=-\cos\theta_{1}\cdot \vec{i}-\sin\theta_{1}\cdot \vec{j}-\frac{\vec{P_{1}}}{r_{1}}.$$

Normal vector $\vec{N_{1}}$ is given by

(10) $$\left\{ \begin{array}{l} \lambda =-\cos\theta_{1}{-x}_{1}\rho \\ \mu =-\sin\theta_{1}-y_{1}\rho \\ \nu =-z_{1}\rho \end{array}\right.,$$

where ρ is the radius of curvature. The expression for P_1_ can be expanded as a multi-order series as follows:

(11) $$\begin{array}{ll} z_{1} & =f(x_{1},y_{1})=\sqrt{r_{1}^{2}-{\left(x_{1}+r_{1}\cos\theta_{1}\right)}^{2}-{\left(y_{1}+r_{1}\sin\theta_{1}\right)}^{2}}\\ & =r_{1}\left[1-\frac{1}{2}{\left(\frac{x_{1}}{r_{1}}+\cos\theta_{1}\right)}^{2}-\frac{1}{2}{\left(\frac{y_{1}}{r_{1}}+\sin\theta_{1}\right)}^{2}\right]+\frac{r_{1}}{8}{\left(\frac{x_{1}}{r_{1}}+\cos\theta_{1}\right)}^{4}-\\ & \frac{r_{1}}{8}{\left(\frac{y_{1}}{r_{1}}+\sin\theta_{1}\right)}^{4}-\frac{r_{1}}{4}{\left(\frac{x_{1}}{r_{1}}+\cos\theta_{1}\right)}^{2}{\left(\frac{y_{1}}{r_{1}}+\sin\theta_{1}\right)}^{2} \end{array}.$$

Differentiating Equation (11) with respect to x_1_ and y_1_, respectively, the normal vector is

(12) $$\vec{N_{1}}=(\frac{\partial z_{1}}{\partial x_{1}},\frac{\partial z_{1}}{\partial y_{1}},-1).$$

Substituting Equation (12) into Equation (10), the intersection point between the incident ray and back surface of prism 1 can be obtained:

(13) $$\left\{ \begin{array}{l} x_{2}=(\frac{\partial z_{1}}{\partial x_{1}}+\cos\theta_{2})\cdot r_{2}\\ y_{2}=(-\sin\theta_{2}-\frac{\partial z_{1}}{\partial y_{1}})\cdot r_{2}\\ z_{2}=r_{2} \end{array}\right..$$

Then, $P_{2}(x_{2},y_{2},z_{2})$ can be expressed with respect to x1 and y1 as follows:

(14) $$x_{2}=-er_{2}-\frac{1}{2}e^{3}r_{2}-\frac{1}{2}ef^{2}r_{2}-\frac{3}{8}e^{5}r_{2}-\frac{3}{4}e^{3}f^{2}r_{2}-\frac{3}{8}ef^{4}r_{2}+\cos\theta_{2}r_{2}$$

and

(15) $$y_{2}=-\sin\theta_{2}r_{2}+fr_{2}+\frac{1}{2}e^{2}fr_{2}+\frac{1}{2}f^{3}r_{2}+\frac{3}{8}e^{4}fr_{2}+\frac{3}{4}e^{2}f^{3}r_{2}+\frac{3}{8}f^{5}r_{2}.\;$$

where $e=\frac{x_{1}}{r_{1}}+\cos\theta_{1},f=\frac{y_{1}}{r_{1}}+\sin\theta_{1}$, based on the paraxial theory and principle of the Rowland circle, the mirror parameters can be calculated, whereas those of prisms can be obtained based on the minimum aberration. Using the method proposed above, the intersection coordinates between the incident ray and each element can be determined in turn, after which the incident and emitted vectors can be provided, and the other initial parameters can be obtained.

## 3. Optomechanical System Design

The proposed spectrometer is aimed at airborne application. Based on previous research and considering the application background [4], the specific technical specifications are listed in Table 1.

To reduce the cost and difficulty of assembly, and the distortion in particular, the telescope is required to have a telecentric image space to achieve pupil matching with the spectrometer. Regarding the specifications, the telephoto system should cover a wide spectrum and include a large relative aperture; the longitudinal aberration, astigmatism, and field curvature need to be corrected. Hence, we selected a double-Gauss structure as the telephoto initial system. Compared to the VNIR band, the types of optical materials that can be used in the SWIR band are limited. Then, we used materials from the Schott catalogs, it was necessary to consider achromatism correction in the design.

The Offner structure, which is a typical layout for a spectrometer, has advantages such as compactness and minimal distortion. Therefore, an Offner structure with a curved prism was selected for the spectrometer. The spectrometer system includes an entrance slit and dispersion elements; the system is non-coaxial, and the slit serves as the entrance for the dispersive optics. The slit allows only a line of the scene into the dispersive optics. Double reflections through the prism enlarge the spectral dispersion width. The materials of three curved prisms are F_silica”.

### 3.1. Initial Spectrometer Calculations

The proposed solution method for the initial design parameters is suitable for curved prism-based spectrometers. The initial structure can be obtained based on the aberration theory and above-deduced mathematical model. The Offner structure consists of three mirrors. According to the paraxial formulas, when the incident and emitted rays are concentric, the aberration is minimum when the junior Seidel coefficient is almost zero. Then, the initial radii and valid object distance of prism 1 can be calculated as follows:

(16) $$l_{1}=r_{p1}(n_{0}+1),r_{p1}=r_{p2}=R_{2},\;R_{1}=2R_{2}.$$

When the object distance and radii of prism 1 are obtained, the incident and emitted vectors of the chief ray can be determined. Based on the Rowland circle, the initial radii of the front and back surfaces of the prism are similar. According to the dispersion characteristics of the curved prism, the incident angle at the front surface of the prism should satisfy a certain relationship. Therefore, the tilt angle of the front surface is determined based on the mathematical model, and the distances between the elements as well as the other parameters of the spectrometer can be obtained based on the aberration and vector theories. Finally, the initial structure of the spectrometer can be obtained, which can be considered as an appropriate starting point for further optimization. The merit function and restrictions are set up to control the aberrations, thereby attaining the required image performance.

### 3.2. Optimization of the Entire System

**Figure 3:**
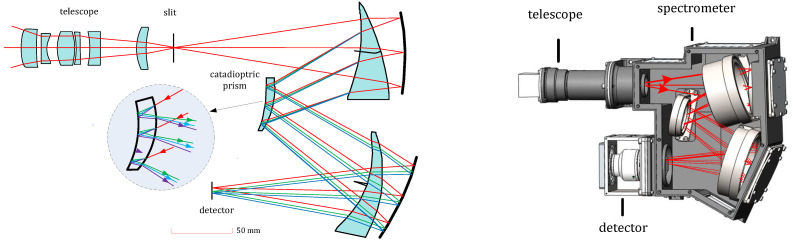
Optical layout and opto-mechanical layout of the system.

**Figure 4:**
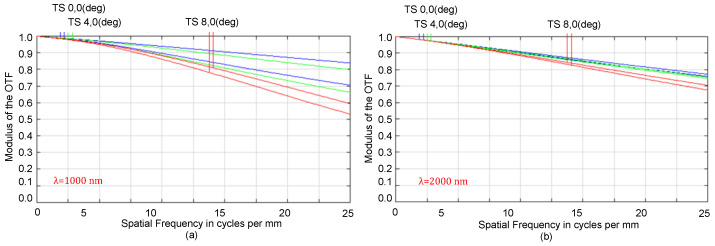
MTF curves of the spectrograph at wavelengths of (**a**) 1000 nm and (**b**) 2000 nm.

The spectrograph system includes a telephoto system, dispersion system, and detectors. Based on the above research and simulation, spherical surfaces are adopted for the optimized system; the telescope and spectrometer are pieced together, and the intermediate and image planes are optimized simultaneously. The optimized opto-mechanical layout and modulation transfer function (MTF) of the typical wavelengths are presented in Figure 3 and Figure 4, respectively. The spectrometer length is 200 mm, representing a decrease of approximately 40% compared to the length of 350 mm in the previous work [2]. Moreover, the distortion of the telephoto is less than 1%, and the dispersion width of the spectrometer is 4.2 mm.

In Figure 4, T and S represent the tangential and sagittal planes, respectively; the red curve represents the MTF of the maximum field of view (FOV); the green curve represents the MTF of the 0.5 FOV; the blue curve represents the MTF of the central FOV; and the MTF curves show that the average values are more than 0.5 for the full spectra.

### 3.3. Tolerance Analysis

Tolerance analysis can elucidate the fabrication and alignment difficulty of the system. We performed tolerance analysis for the spectrograph. The tolerance items included the surface/element tilt, surface/element decenter, thickness, and surface irregularity. These values are loose in the actual system. The diffraction MTF at 1800 nm wavelength was applied as the nominal tolerance criterion. Table 2 shows the six items that have the greatest effects on the MTF. The analysis results are as follows.

As seen in Table 2, the first three terms are related to the curved prisms of the spectrometer. The last terms pertain to the telescope, which change the MTF slightly, so the accuracy of processing and assembly of the prisms should be guaranteed.

### 3.4. Prototype Fabrication and Assembly

**Figure 5:**
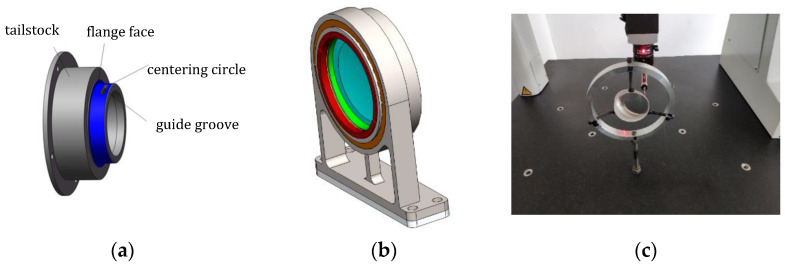
Mechanical design of the catadioptric prism and prism testing process: (**a**) Centering tailstock of the prism frame; (**b**) components of the assembled structure; (**c**) prism testing.

A curved prism is used as the special dispersion element to obtain spectral data; the spherical centers of the front and rear surfaces are not on the same optical axis. Conventional lens processing and coating technology are not applicable. A highly accurate customized fixture is developed to ensure the tilt angles of the prism. A certain deviation between the rotation axis of the optical element and the motion center of the tool is set to generate the prism parameters. The main parameters of a curved prism are the tilt angles and the radii of the front and back surfaces, which have immediate impacts on the dispersion performance; therefore, the test accuracy is crucial. According to the tolerance analysis, the accuracy of the catadioptric prism is sensitive; therefore, the requirement of installation accuracy is rigid. However, as the shape of the curved prism is different from that of the traditional lens, the use of a common clamp in the structure is not suitable. Hence, a special structure must be designed to ensure the accuracy and stability of the installation. Figure 5a,b show the flexible mount of a prism. The outgoing surface and outer cylinder of the prism are taken as the installation references, and a centering tailstock is designed for the prism frame. The centering processing is used to ensure coaxiality between the outer circle of the metal frame and the prism, as well as the perpendicularity between the end face of the frame and the optical axis of the prism. The guide groove is used to rotate the relative position between the components and the frame, ensuring the assemble accuracy of the elements. In this study, a three-coordinate measuring machine with 2 mm ruby probes was used to test the outer circle and the front and back surfaces of the curved prism. The relative positions of the optical axis and spherical centers of the curved prism were obtained by fitting the test data. Figure 5c displays the optical test process. The test results satisfy the tolerance specifications.

**Figure 6:**
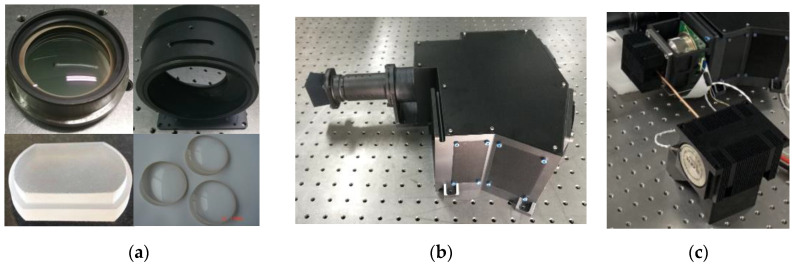
Spectrometer prototype: (**a**) Optical and mechanical elements; (**b**) prototype; (**c**) detector.

The fabricated elements are shown in Figure 6a, including the prisms, mirrors, and spherical lens. After the optical elements were fabricated and tested, the spectrograph prototype was adjusted. The prototype is shown in Figure 6b. The alignment of the telephoto system is easy, whereas that of the spectrometer is difficult. The alignment of the prototype involves four steps: alignment of the telephoto system, matching of the slit with the spectrometer, assembly of the slit and spectrometer, and alignment of the opto-mechanical structure and detector. The slit location relative to the telephoto ensures accurate input into the spectrometer. The distance of the slit separate from the spectrometer will lead to a noncoplanar deviation between the spectral and spatial images. Therefore, the adjustment device is constructed to guarantee that the target imaged by the telephoto system is coplanar with the slit. This device includes a collimator, microscope, lamp source, and detector. By adjusting the gasket thickness, the image of the given target is aligned and made coplanar with the slit; the slit is imaged by the spectrometer on the detector and dispersed into multiple spectral bands.

## 4. Prototype Performance Tests

### 4.1. Spatial Performance Optical Tests

**Figure 7:**
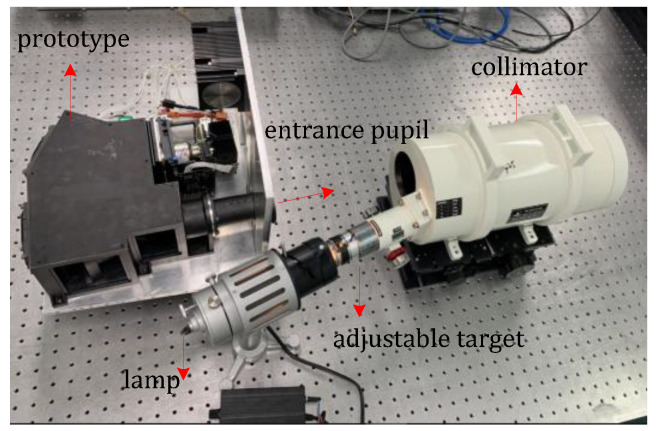
Experimental setup.

The critical parameters for measuring the performance of the prototype are the spatial and spectral resolutions. Here, we used customized targets to test the spatial resolution of the prototype. According to the spatial resolution, focal length of the system, focal length of the collimator, and size of the detector pixel, a target with distinguished widths and directions was designed and calculated. We used a halogen lamp as the light source because it covered the SWIR spectrum. For the laboratory experiment, the test equipment included a customized target, collimator, halogen lamp, prototype, and computer. The experimental setup is shown in Figure 7.

**Figure 8:**
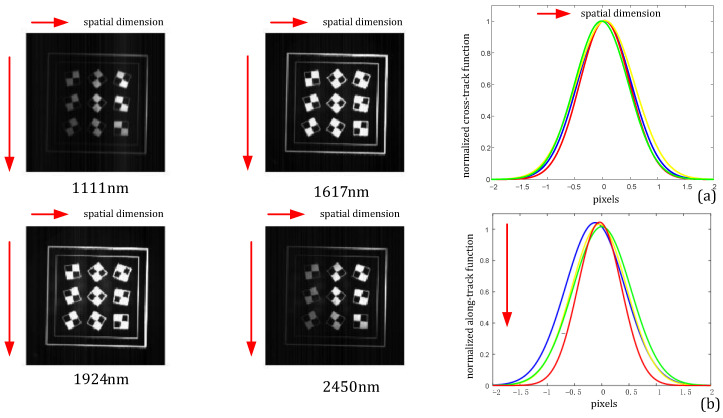
(**a**) Cross-track spatial response functions of spatial pixels for the 1111, 1617, 1924, and 2450 nm wavelengths; (**b**) along-track spatial response functions for the 1111, 1617, 1924, and 2450 nm wavelengths.

The target was located on the object plane of the reflective collimator and illuminated by the halogen lamp; the parallel beam from the collimator was incident on the prototype. The target was customized according to the specifications and consisted of several sets of black-white intersection square lines. The spectrograph system was adjusted to collect the image of the target at the center of the detector. The spatial characteristics included the cross-track and along-track spatial response functions. In the test, a target placed at the focal plane of the collimator was scanned to obtain pushbroom images of different FOVs, and four typical wavelengths are shown in Figure 8a. The FWHW indicates the contrast of light and dark. The FWHWs of cross-track spatial functions are about 1.2 pixels. The along-track spatial functions are depicted in Figure 8b, and the full widths at half-maximum (FWHMs) of four typical wavelengths are about 1.3 pixels. The values of the other off-axis FOVs are similar. These findings prove that the spatial resolution of the spectrograph system satisfies the design requirements and reliability.

### 4.2. Spectral Performance Optical Tests

**Figure 9:**
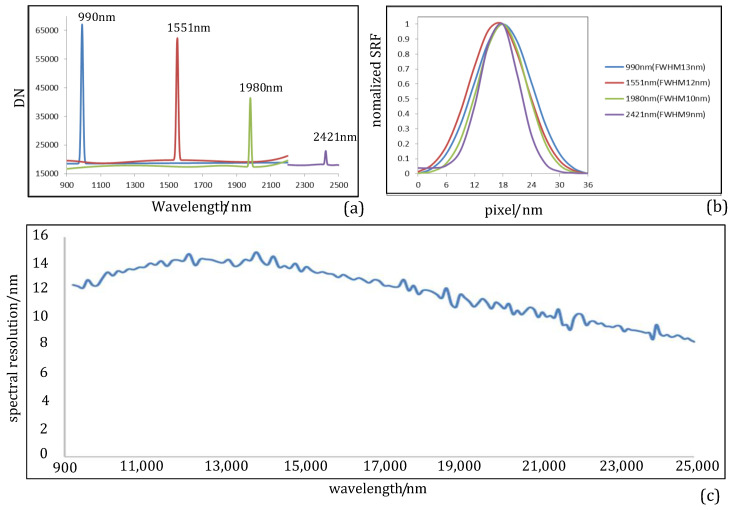
(**a**) SRFs of typical wavelengths; (**b**) FWHM curve of typical wavelengths; (**c**) measured spectral resolutions of the prototype in the SWIR spectrum.

The spectral response function (SRF) was obtained by applying the previously used off-axis collimator and a grating monochromator (HRS-500). Typical spectral lines of the source were selected to measure the spectral bandwidth of the prototype. The contour lines of the SRF were then described, and the spectral resolution was obtained. The monochromator used for the test had a focal length of 500 mm; a halogen lamp was selected as the source; and the output beam was varied continuously across the entire spectral band. To obtain highly accurate spectral data, a monochromator with a slit of 35 μm and a grating of 300 g/mm was used. A monochrome beam was incident on the spectrograph system, and the slit images of the sampling spectral lines were detected. A column of image data was drawn, and the SRF sampling curves are depicted in Figure 9a,b, where the *x*-axis denotes the SWIR spectrum range and the *y*-axis denotes the relative digital number (DN). SRFs corresponding to wavelengths of 990, 1551, 1980, and 2421 nm are presented from left to right. The FWHM of the entire spectrum is shown in Figure 9c, which reveals that the spectral resolution varies from 8.3 to 14.42 nm, whereas the variation in the spectral dispersion is approximately uniform. These findings are in almost exact agreement with the theoretical spectral resolution.

**Figure 10:**
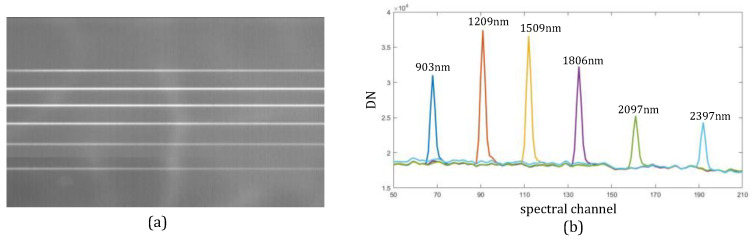
(**a**) Slit images corresponding to wavelengths of 903, 1209, 1509, 1806, 2097, and 2397 nm and (**b**) radiation intensities of different channels.

To test the spectral performance of the prototype in detail, radiation calibration of the detector and spectral calibration was completed. Therefore, we selected the slit images of the full FOV with sampled isolated spectral lines to test the performance. The images on the detector and the radiation intensity of the images are shown in Figure 10, where the *x*-axis denotes the SWIR spectrum channels and the *y*-axis denotes the relative DN. The above figure helps confirm the positions of different wavelengths on the detector and proves the spectral reliability of the design.

### 4.3. External Imaging Tests

**Figure 11:**
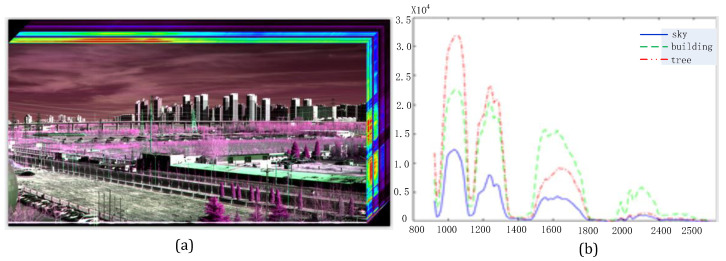
Imaging of external vision: (**a**) data cube of color images, (**b**) spectral reflective DNs of three different objects.

To test the performance of the prototype under various outdoor conditions, we placed the prototype on a swivel machine and scanned external scenes at a matching speed. The data cubes obtained from the test provided reference information for the subsequent data processing. Figure 11 provides an image with 2500 frames of continuous far vision sweeping; the corresponding images of the typical wavelengths and DNs of different objects were obtained and selected in order from short to long wavelength. Trees, houses, and the outlines of distant mountains are clearly observed, demonstrating the imaging ability of external scenes under actual conditions. The SNR was calculated under external imaging, and the analysis result is more than 220, which satisfies the application requirements. The SNR can be improved by extending the exposure time or using a reflection telescope with high efficiency.

Figure 11 displays the data cube images and spectral characteristics of a tree, a building, and the sky at the given positions. In Figure 11b, where the *x*-axis denotes the wavelength and the *y*-axis denotes the reflective characteristics of the corresponding objects across the spectrum, three obvious absorption peaks are found near 1150, 1400, and 1900 nm, which are the characteristics of the atmosphere in the SWIR spectral band. The tree exhibits two peaks, located at 1200 and 1400 nm. The other locations are almost smooth.

## 5. Conclusions

In this study, a design method using a catadioptric prism was proposed, and the mathematical model of a curved prism-based spectrometer was presented. We introduced and elaborated the design methods in detail. The proposed method was used to construct the initial structure of a curved prism-based spectrometer, simplifying the optical design process and reducing the volume of the system, whose surfaces were all spherical. To verify the proposed method, a SWIR imaging spectrometer was designed and developed, and the performance of the prototype was tested systematically. The obtained spectral resolution varied from 8.3 to 14.42 nm in the SWIR spectral band. The test results demonstrate that the spectral and spatial performances are consistent with the theoretical design. Under the same specifications, compared to the reported spectrometer length of 350 mm, the length of the spectrometer in this study was only 200 mm, representing a decrease of 40%, and the dispersion width was 4.2 mm. The volume was compact and beneficial for the satellite, and the production and alignment costs were low. The proposed method provides a starting point for initial structure construction and volume reduction and can serve as a reference for the design of related off-axis systems.

## Figures and Tables

**Table 1:** Specifications of the spectrograph system.

Parameter	Value	Units
spectrum	950–2500	nm
F number	3	-
field-of-view	16	degree
angle resolution	0.27	mrad
pixel size	24 × 32	μm
spectral band	132	-

**Table 2:** Sensitive terms influencing the system in the tolerance analysis.

Tolerance Item	Element	Given Value	Change in MTF
Surface tilt	Prism 2	−0.03–0.03	0.055
Element decenter (mm)	Prism 3	−0.03–0.03	0.048
Surface tilt (°)	Prism 1	−0.03–0.03	0.04
Element tilt (°)	Mirror 1	−0.03–0.03	0.035
Surface decenter (mm)	Lens 2 (telescope)	−0.04–0.04	0.032
Element decenter (mm)	Lens 1 (telescope)	−0.04–0.04	0.026

## Data Availability

Not applicable.
